# The Impact of Dyspepsia on Symptom Severity and Quality of Life in Adults with Headache

**DOI:** 10.1371/journal.pone.0115838

**Published:** 2015-01-28

**Authors:** Mei-Ling Sharon Tai, Norbelinda Norhatta, Khean Jin Goh, Foong Ming Moy, Ramanujam Sujarita, Azman Ahmad Asraff, Qin Zhi Lee, Jiun Hoong Ng, Eugene Choon Li Tan, Sanjiv Mahadeva

**Affiliations:** 1 Division of Neurology, Department of Medicine, Faculty of Medicine, University Malaya, Kuala Lumpur, Malaysia; 2 Department of Social and Preventive Medicine, Faculty of Medicine, University Malaya, Kuala Lumpur, Malaysia; 3 Department of Primary Care, Faculty of Medicine, University Malaya, Kuala Lumpur, Malaysia; 4 Division of Gastroenterology, Department of Medicine, Faculty of Medicine, University Malaya, Kuala Lumpur, Malaysia; University Hospital Llandough, UNITED KINGDOM

## Abstract

**Background:**

Dyspepsia and headache frequently co-exist, but the clinical implication of this association is uncertain. We planned to examine the prevalence and impact of dyspepsia in adults with headache.

**Methods:**

A cross-sectional study was conducted in a secondary care setting. Clinical, psychological and health-related quality of life (HRQOL) data were compared between subjects with headache and controls (non-headache subjects). The impact of dyspepsia was analysed further in subjects with headache alone.

**Results:**

280 subjects (93 cases with headache and 187 matched controls) were recruited. The following baseline characteristics of subjects were as follows: mean age 45.0±17.3 years, 57.0% females and ethnic distribution—Malaysian = 45 (48.4%), Chinese n = 24 (25.8%) and Indians n = 24 (25.8%). Headache sub-types among cases with headache were as follows: tension-type headache (TTH) n = 53 (57.0%) and migraine n = 40 (43.0%). Dyspepsia was more prevalent in cases with headache compared to controls (25.8% vs 12.8%, p = 0.011), and headache was independently associated with dyspepsia (OR 2.75, 95% CI 1.39–5.43). Among cases with headache, there was a trend towards a higher prevalence of dyspepsia in those with migraine (27.5%) compared to TTH (24.5%). Subjects with headache and dyspepsia, compared to those with headache alone, had a greater severity of headache symptoms (63.67±22.85 mm vs 51.20 ±24.0 mm VAS, p = 0.029). Overall HRQOL scores were lower in headache subjects with dyspepsia (EQ-5D summary score 0.82±0.18 vs 0.90 ±0.16, p = 0.037 and EQ-5D VAS 62.08±17.50 mm vs 72.62 ±18.85 mm, p = 0.018), compared to those without dyspepsia.

**Conclusion:**

Dyspepsia is associated with more severe headache symptoms and results in a lower HRQOL in patients with headache.

## Introduction

Headache and gastrointestinal symptoms are common in the community [1]. Previous studies have shown that the prevalence of headache in the community range from 17% to 21% [2, 3]. Similarly, upper gastrointestinal symptoms, particularly dyspepsia, have been reported to be prevalent in 24.3%–38.1% of the adult population [4]. Recent reports have suggested that these common conditions may be associated [5]. A higher prevalence of headache has been reported in adults who complained of various gastrointestinal symptoms such as reflux symptoms, diarrhoea, constipation and nausea [6]. Whilst more dyspepsia has been observed in migraine sufferers [7], a higher prevalence of migraine has been described in adults with dysmotility-like dyspepsia [5].

Several pathophysiological mechanisms have been suggested to explain the association between headache, particularly migraine, and dyspepsia. An abnormal visceral mechano-sensory vagal function [8, 9] and an excess of certain neuropeptides, have been described in both migraine and dyspepsia [10, 11]. The neuropeptide calcitonin gene-related peptide (CGRP) is known to increase during acute episodes of migraine [12]. CGRP is also an important neurotransmitter of the inhibitory sensory neuron and plays a role in visceral afferent nerve sensitization of the gastrointestinal tract, which can result in functional dyspepsia symptoms [13, 14].

Whilst the association of headache and dyspepsia is recognised, the impact of dyspepsia in patients with headache has not previously been studied. In particular, there is a paucity of data on health-related quality of life (HRQOL) consequences due to the co-existence of both conditions in a patient. We therefore conducted a study to provide more data in this area, in particular, to determine the prevalence of dyspepsia and examine its’ impact on symptom severity and HRQOL in a cohort of Asian subjects with headache.

## Methodology

### Study subjects

A cross-sectional study was conducted in a tertiary institution, the University Malaya Medical Centre, from February 2012 until September 2012. The study was approved by the Institutional Ethics Committee of University Malaya Medical Centre. Cases consisted of subjects, aged ≥ 18 years, with headache at least once per month for more than three months attending the specialist neurology outpatient clinic. Patients with headache secondary to intracranial mass lesions and secondary headache were excluded. Patients with headache on aspirin or other non-steroidal anti-inflammatory drugs (NSAIDs), a known history of gastroesophageal reflux or peptic ulcer disease were also excluded.

At the same time, control subjects who were age, gender and ethnicity matched with the study subjects, were recruited. They were non-related carers and friends who travelled with patients. Written informed consent was obtained from all the study participants or their legally acceptable representatives.

### Study design

All consenting subjects were interviewed with a structured questionnaire, which included information on relevant clinical and demographic parameters, details of headache characteristics, details of GI symptoms (see below), features of anxiety and/or depression (see below) and HRQOL (see below). The diagnosis of the headache was based on the International Headache Society (IHS) Criteria (ICHD III) [15]. The International Headache Society (IHS) classification, International Headache Criteria II (ICHD-III) was used to classify the headache subtypes [15]. The severity/ intensity of headache symptoms was based on a visual analogue scale (VAS), which has previously been shown to be valid for this purpose [16].

### Specific instruments

The presence of dyspepsia was confirmed by a locally translated and validated version of the Leeds Dyspepsia Questionnaire [17]. The LDQ is an eight item symptom-based questionnaire detailing the frequency and severity of various upper gastrointestinal symptoms [18]. The upper gastrointestinal symptoms comprise of dysphagia, belching, upper abdominal pain/discomfort, nausea, vomiting heartburn, regurgitation, and post-prandial distension/early satiety. A score ranging from 0 to 40 can be calculated in the LDQ based on item frequency, and a cut-off of 11/40 has previously been shown to be diagnostic of significant dyspepsia in Malaysian adults [17]. The various subtypes of dyspepsia according to Rome II criteria were also documented based on the predominant symptom in the LDQ [19].

The Hospital Anxiety Depression Scale (HADS) [20] and the Hamilton Depression Scale [21] were used to assess for depression and anxiety in study subjects. The HADS consists of seven questions on anxiety and seven questions on depression, with relevant scoring for mild, moderate and severe disease. A translated version of the HADS has previously been validated and shown to be reliable in our local population [22]. The Hamilton Depression scale consists of 17 items which are rated and has relevant scores for mild, moderate and severe depression as well.

HRQOL was assessed using the EQ-5D (Euroqol). The EQ-5D comprises five questions on major domains of HRQOL, i.e. mobility, self-care, pain, usual activities and psychological status with three possible answers for each item (1 = no problems, 2 = moderate problems, 3 = severe problems) [23]. An overall utility score is calculated based on these domains, with a score ranging from 0 (worse health scenario) to a maximum of 1.0 (best health scenario). An additional visual analogue scale (VAS, scale 0–100) is used to assess general health status with 100 indicating the best health status. Malaysian English and Malaysian Malay versions of the EQ-5D were developed by the EuroQoL Group: 2005 (original developers) using their standard translation and linguistic validation process [24] and have been validated for use in Malaysia [25].

### Statistical analysis

All descriptive statistics were done using Statistical Package for Social Sciences, SPSS (Version 16.0, SPSS Inc., Chicago, USA). Chi square test (or Fisher’s exact test) were used to analyse categorical data, whilst an independent sample T-test was used for continuous data analysis. Multivariate analysis was carried out to identify independent predictors of dyspepsia, using a logistic stepwise regression model. Risk associations were reported as odds ratios (OR) with a 95% confidence interval (CI). A p value of < 0.05 (two-tailed p value) was taken as statistical significance.

## Results

During the study period, 93 subjects with headache and 187 controls were recruited at the specialist neurology clinic at University Malaya Medical Centre. Table 1 highlights the demographic and clinical comparisons between subjects with headache (cases) and those without headache (controls). Patients with headache and controls were matched for age (45.0±17.3 years headache vs 42.1±15.4 years control, p = 0.15), gender (57% females headache vs 66.3% females controls, p = 0.15) and ethnicity (Table 1).

**Table 1:** Baseline characteristics of subjects with headache and non-headache controls.

	**Patients, n = 93**	**Controls, n = 187**	**p value**
**Age** (mean±SD)	45.0±17.3	42.1±15.4	0.15
**Gender** (n, %)			
Male	40 (43%)	63(33. 7%)	0.15
Female	53 (57%)	124(66.3%)	
**Ethnic group** (n, %)			
Malay	45 (48.4%)	102 (54.5%)	0.61
Chinese	24 (25.8%)	41 (21.9%)	
Indian	24 (25.8%)	44 (23.5%)	
**BMI** (kg/m2)			
(mean±SD)	25.2±5.1	24.4±4.7	0.23
**Marital status** (n, %)			
Single	21(22.6%)	51(27.3%)	0.33
Married	64(68.8%)	116(62.0%)	
Widow/widower	6(6.4%)	19(10.2%)	
Divorced	2(2.2%)	1(0.5%)	
**Educational Level** (n, %)			
Primary	5(5.4%)	13 (7.0%)	0.22
Secondary	12(12.9%)	29(15.5%)	
Pre-university	43(46.2%)	70(37.4%)	
Diploma	23(24.7%)	32(17.1%)	
Tertiary	9(9.7%)	38(20.3%)	
**Smoking** (n, %)			
Yes	10(10.8%)	13(7.0%)	0.003
No	83(89.2%)	174(93.0%)	
**Alcohol** (n, %)			
Yes	0	5 (2.7%)	0.002*
No	93 (100%)	182(97.3)	
**Concomitant medical illness** (n, %)			
**Cardiovascular**	21(22.6%)	21 (11.2%)	0.036
**Metabolic**	16(17.2%)	19(10.2%)	0.12
**Respiratory**	14(15.1%)	21(11.3%)	0.44
**Renal/Genitourinary tract**	1 (1.1%)	2(1.1%)	1.00
**Rheumatological Disease**	3(3.3%)	3(1.6%)	0.40
**Dermatological Disease**	2(2.2%)	4(2.2%)	1.00
**Neurological Disease**	1(1.1%)	1(0.5%)	1.00
**Dyspepsia** (n, %)	24 (25.8%)	24(12.8%)	0.011
**HADS-D: moderate and severe** (n, %)	11 (11.8%)	19 (10.2%)	0.69
**HADS-A: moderate and severe** (n, %)	18 (19.4%)	32(17.1%)	0.74
**Hamilton score**	4.39±4.80	4.19±5.34	0.77

*Likelihood Ratio Chi-square test

There were more smokers among cases (10.8% vs 7.0%) and a slightly greater amount of alcohol intake in controls (2.7% vs 0%) (Table 1). Cases with headache additionally had more co-morbidities in terms of cardiovascular (22.6% vs 11.2%) and metabolic (17.2% vs 11.2%) diseases.

### Headache characteristics

Headache characteristics of the study subjects are shown in Table 2. Most of the patients had a headache onset at anytime of the day. About one-third of the patients had headache for ≥ 15 days in a month. In terms of lateralization of headache, most patients complained of unilateral and alternating unilateral headache. The most frequently affected area was the temporal area and approximately half had a throbbing headache. Headache sub-types were as follows: tension-type headache (TTH) n = 53 (57.0%) and migraine n = 40 (43.0%). Among the migraine sub-types, 26 (65.0%) patients had migraine without aura and 26 (65.0%) had migraine without aura. The TTH sub-type group had the following characteristics: frequent TTH n = 31 (58.5%), infrequent TTH n = 6 (11.3%) and chronic TTH n = 16 (30.2%). The mean score of the visual analogue scale (VAS) for headache was 54.4±24.2 mm. VAS for headache intensity was used for current headache symptoms. More than half of the patients took paracetamol for headache, but a significant number of patients (37.6%) also used traditional medicated oil for analgesia.

**Table 2:** Headache characteristics of patients.

**Characteristics**	**n = 93**
**TIME OF ONSET** (n, %)	
On rising	18(19.4%)
Afternoon/evening	30(32.3%)
Night	10(10.8%)
Anytime	35 (37.6%)
**FREQUENCY OF HEADACHE** (n, %)	
Every day	23(8.2%)
Every other day	4(1.4%)
Once to three times weekly	16(5.7%)
Two to four times per month	34(12.1%)
Once monthly	16(5.7%)
**NUMBER OF HEADACHE EPISODES IN A MONTH** (n, %)	
Headache < 15 days/month	66 (71.0%)
Headache ≥15days/month	27 (29.0%)
**SITE OF PAIN** (n, %)	
Frontal	30(32.3%)
Temporal	31(33.3%)
Occipital	11(11.8%)
Vertex	1(1.1%)
Whole head	20(21.5%)
**LATERALITY OF PAIN** (n, %)	
Unilateral	29 (31.2%)
Bilateral	36 (38.7%)
Alternating unilateral	26(28.0%)
Orbital	2(2.2%)
**CHARACTER OF HEADACHE** (n, %)	
Throbbing/pulsating	51(54.8%)
Sharp/stabbing	11(11.8%)
Tightness/pressing	29(31.2%)
Pricking	2(2.2%)
**INTENSITY OF HEADACHE** (n, %)	
Mild	21 (22.6%)
Moderate	43 (46.2%)
Severe	29 (31.2%)
**TRIGGER FACTORS** (n, %)	
Stress	55(59.1%)
Lack of sleep	44(47.3%)
Weather	30(32.3%)
Sun exposure	26(28%)
Too much sleep	16(17.2%)
Certain food and drinks	16(17.2%)
Missing meal	16(17.2%)
Menstruation	7(7.5%)
**HEADACHE SUBTYPES** (n, %)	
Frequent TTH	31(33.3%)
Chronic TTH	16(17.2%)
Infrequent TTH	6(6.5%)
Migraine without aura	26(28%)
Migraine with aura	14(15.1%)
**MEDICATION USED TO RELIEVE HEADACHE** (n, %)	
**Acute treatment**	
Paracetamol	61 (65.6%)
Tramadol	5 (5.4%)
Ergotamine	5(5.4%)
Sumatriptan	3(3.2%)

### Prevalence of dyspepsia in headache

Dyspepsia was more prevalent in subjects with headache compared to non-headache controls (25.8% vs 12.8%, p = 0.011). However, dyspepsia prevalence did not differ among the major headache types (27.5% migraine vs 24.5% TTH, p = 0.81). Six patients who had migraine with aura and five patients who had migraine without aura complained of dyspepsia. (p = 0.15) There were no statistically significant differences between various types of headache and dyspepsia (p = 0.84). Similarly, dyspepsia sub-types (according to the Rome II criteria) were not different between headache categories as follows: ulcer-like (18.2% migraine vs 23.1% TTH), motility-like (36.4% migraine vs 46.1% TTH) and reflux-like (45.4% migraine vs 30.8% TTH).

The association of dyspepsia with headache was explored further in a multivariate regression model with various recognised variables (Table 3). Predictive factors for dyspepsia in our subjects were found to include headache (OR 2.75) and anxiety (OR 3.52) only, indicating a strong link between headache and dyspepsia.

**Table 3:** Multivariate analysis of predictors of dyspepsia.

**Factors**	**Dyspepsia**		**Unadjusted OR**	**95% CI**	**Adjusted OR**	**95% CI**	**p value**
	**Yes (n = 48)**	**No (n = 232)**					
**Age** (n, %)							
< 50 years	33 (18.9)	142(81.1)	1.00		1.00		
≥ 50 years	15 (14.3)	90(85.7)	0.71	0.37–1.40	0.66	0.32–1.40	0.28
**Gender** (n, %)							
Male	16(15.5)	87(84.5)	1.00		1.00		
Female	32(18.1)	145(81.9)	1.20	0.62–2.31	1.63	0.79–3.37	0.19
**Smoking** (n, %)							
No	45(17.5)	212(82.5)	1.00				
Yes	3 (13.0)	20 (87.0)	0.71	0.20–2.48	-	-	-
**Alcohol** (n, %)							
No	48 (17.5)	227 (82.5)					
Yes	0 (0)	5 (100)	NA	NA	-	-	-
**Anxiety** (n, %)							
No	29 (12.6)	201 (87.4)	1.00		1.00		
Yes	19 (39.1)	31 (61.9)	4.37	2.16–8.85	3.52	1.67–7.44	0.001
**Depression** (n, %)							
No	37 (14.8)	213 (85.2)	1.00		1.00		
Yes	11 (36.7)	19 (63.3)	3.33	1.47–7.57	2.41	0.99–5.91	0.05
**Headache** (n, %)							
No	24 (12.8)	163(87.2)	1.00		1.00		
Yes	24 (25.8)	69 (74.2)	2.36	1.36–4.44	2.75	1.39–5.43	0.004

### Impact of dyspepsia on headache

Differences in the clinical severity of headache and EQ-5D parameters were explored between headache subjects with and without dyspepsia. Subjects with headache and dyspepsia had a higher VAS-pain score compared to those with headache alone (63.67±22.85 mm vs 51.20 ±24.00 mm VAS, p = 0.029). Table 4 highlights the differences in HRQOL domains between headache subjects with and without dyspepsia. Headache subjects with dyspepsia reported more problems with “pain/ discomfort” (62.5% vs 34.8%, p = 0.029) and “anxiety” (25.0% vs 7.2%, p = 0.03) compared to those with headache alone (Table 4). Globally, subjects with headache and dyspepsia had a lower EQ-5D utility score (0.82±0.18 vs 0.90±0.16, p = 0.037) and a lower EQ-5D VAS measurement (62.08±17.50 mm vs 72.62 ±18.85 mm, p = 0.018) compared to subjects with headache alone.

**Table 4:** HRQOL differences between headache with and without dyspepsia.

**EQ-5D (n, %)**	**Headache and dyspepsia (n = 24)**	**Headache alone(n = 69)**	**p value**
**Mobility**			
No problem	20(83.3%)	63(91.3%)	
Problem	4(16.7%)	6(8.7%)	0.28
**Self-caring**			
No problem	24(100%)	65(94.2%)	
Problem	0	4(5.8%)	0.57
**Activities**			
No problem	22(91.7%)	65(94.2%)	
Problem	2(8.3%)	4(5.8%)	0.65
**Pain or discomfort**			
No problem	9(37.5%)	45(65.2%)	
Problem	15(62.5%)	24(34.8%)	0.029
**Anxiety**			
No problem	18(75%)	64(92.8%)	
Problem	6(25%)	5(7.2%)	0.03
**EQ-5D VAS**	62.08±17.50	72.62±18.85	0.018
**EQ-5D score**	0.82±0.18	0.90±0.16	0.037

## Discussion

This study has affirmed several observations between dyspepsia and headache. Compared to non-headache controls, subjects with headache had a significantly higher prevalence of dyspepsia. Among subjects with headache, a higher prevalence of dyspepsia (27.5%) was observed among those with migraine in this study. Our findings appear to concur with previous reports demonstrating a particular predilection for dyspepsia among patients with migraine (up to 60% in the study by Kurth et al) [7]. A higher frequency of smoking and co-morbidities, known risk factors for dyspepsia [26], among subjects with headache may have resulted in more dyspepsia. However, we performed a multivariate regression analysis which demonstrated an independent association between dyspepsia and headache. This association between headache and dyspepsia was independent of the presence of anxiety and depression, well recognised associations for both these condition [27–30].

The impact of dyspepsia on symptom severity in headache is a novel finding in this study. Headache subjects with dyspepsia had more severe symptoms compared to those cases without dyspepsia. A possible explanation may be due to a heightened common pathophysiological mechanism, such as visceral sensitisation, which may lead to more severe symptoms in cases with dual pathology compared to just headache alone.

In addition to an impact on symptom severity, we have demonstrated that dyspepsia resulted in a lower HRQOL in subjects with co-existing headache and dyspepsia. Both headache and dyspepsia, on their own, have been reported to be associated with a lower HRQOL among sufferers in the community [26, 31]. It is not entirely surprising then, that the combination of both conditions in adults, as opposed to just headache alone, would result in a greater HRQOL impairment. Examining the specific domains of HRQOL within the EQ-5D instrument, it is apparent that greater problems of “pain” and “anxiety” were reported in adults with co-existing dyspepsia and headache, compared to those with headache alone. Although not conclusive, it can be assumed that an increased headache symptom severity and possible psychological disturbance may have contributed to the lower HRQOL in subjects with dyspepsia and headache.

A previous large endoscopy-based study examined the association between dyspepsia and migraine [5]. Among 378 patients with dyspepsia and 310 controls, Meucci et al showed no difference in the prevalence of migraine. However, they were able to observe a greater prevalence of migraine among subjects with motility-like dyspepsia, compared to ulcer-like and reflux-like dyspepsia. Our study did not demonstrate a similar association between headache and dyspepsia sub-types for several possible reasons. We included various types of headache in this study, and not just migraine. The proportion of subjects with migraine alone was fewer than in Meucci’s study, which may have lead to a Type II statistical error in our findings. Finally, our definitions of dyspepsia may have differed. The inclusive definition of dyspepsia used in the LDQ, which include symptoms of GERD as well, may appear to conflict with the Rome process [32]. However, the Rome definition of dyspepsia, and its requisite of excluding any symptoms of GERD, has been criticised for excluding many patients with genuine FD in both Asian [33] and non-Asian communities [34]. It has even less applicability for uninvestigated dyspepsia in the community, where both reflux symptoms and epigastric pain commonly coexist.

There were several limitations to this study. The study was conducted in a secondary care setting, with the selection bias of subjects with more severe symptoms or greater anxiety, leading to an increased health-care seeking behaviour. Hence, the findings from this study may not be relevant to adults with less complicated headache residing in the community. However, due to the structure of the health-care system in urban Malaysia, many patients are able to consult specialists in secondary/ tertiary institutions without prior primary care visitation [1]. Hence, it is likely that some of the subjects recruited in this study may be representative of headache cases in the community. Several important factors which may have been relevant to the development of dyspepsia, such as dietary intake and *H. pylori* infection, were not measured in this study.

## Conclusion

We conclude that dyspepsia is strongly associated with headache, particularly in cases with migraine. Compared to cases without dyspepsia, headache cases with dyspepsia have more severe symptoms and a lower HRQOL. The impact of dyspepsia in headache has clinical implications – i.e. treatment of dyspepsia (and its’ underlying causes) may improve HRQOL in such patients.

## Supporting Information

S1 Data Data for the study. (XLS)
